# 

**Published:** 1911-03

**Authors:** 


### THE BRISTOL

### Medico-Chirurgical Journal.

A JOURNAL OF THE MEDICAL SCIENCES FOR THE WEST OF ENGLAND AND SOUTH WALES

*PUBLISHED UNDER THE AUSPICES OF*

*THE BRISTOL MEDICO-CHIRURGICAL SOCIETY.*

EDITOR:

R. SHINGLETON SMITH, M.D., B.Sc.,

WITH WHOM ARE ASSOCIATED

J. MICHELL CLARKE, M.A., M.D., J. LACY FIRTH, M.S., M.D.
AND JAMES SWAIN, M.S., M.D.

ASSISTANT-EDITOR:

P. WATSON WILLIAMS, M.D.

EDITORIAL SECRETARY:

J. A. NIXON, B.A., M.B.

---

"Scire est nescire, nisi id me

Scire alius sciret."

---

LIBRARY

SURGEON GENERAL'S OFFICE

MAY 2 1912

211588

*VOL. XXIX.*

BRISTOL: J. W. ARROWSMITH LTD.
LONDON: J. & A. CHURCHILL, 7 GREAT MARLBOROUGH STREET.
1911.

WI
BR323

J. W. Arrowsmith Ltd

Publishers Bristol

33756

### CONTENTS OF VOLUME XXIX.

Page

The Long Fox Lecture: Cerebro-Spinal Syphilis. By J. MICHELL CLARKE, M.A., M.D. Cantab., F.R.C.P. .. .. .. .. 1

Defects in the Visual Field. By F. RICHARDSON CROSS, M.B. Lond., F.R.C.S. .. .. .. .. .. .. .. .. 33

The Nature and Treatment of Chorea. By CAREY F. COOMBS, M.D. Lond., M.R.C.P. .. .. .. .. .. .. 51

On the Resection of the Posterior Spinal Nerve Roots. By ERNEST W. HEY GROVES, M.S. Lond., F.R.C.S. .. .. 105

Some Observations on School Hygiene. By J. O. SYMES, M.D. Lond., D.P.H. .. .. .. .. .. .. .. .. 109

Design for an Elementary Radiographic Camera. By WILLIAM COTTON, M.D. Edin. .. .. .. .. .. .. .. 118

The Medical Aspect of Boswell's *Life of Johnson*, with some Account of the Medical Men mentioned in that Book. By BERTRAM M. H. ROGERS, M.D., B.Ch., B.A. Oxon. .. .. .. .. 125

Auricular Fibrillation. By C. E. K. HERAPATH, M.D. Lond. .. 148

Furunculosis. By L. SHINGLETON SMITH, M.B. Cantab. .. .. 157

Four Cases of Cysts in the Neck of Similar Character, and Possibly Developed in connection with Branchial Clefts. By CHARLES A. MORTON, F.R.C.S. .. .. .. .. .. .. .. 160

A Case of Ataxia in a Child aged three and a half years. By EDWARD CECIL WILLIAMS, B.A., M.B. Cantab. .. .. .. .. 163

Medical Organisation and the Growth of the Medical Sciences in the Seventeenth Century, Illustrated by the Lives of Local Worthies. By GEORGE PARKER, M.A., M.D. Cantab. .. .. 201

The End-Results of Operations on the Stomach and Duodenum. By A. RENDLE SHORT, B.Sc., M.D. Lond., B.S., F.R.C.S. .. 220

CONTENTS.

*Page*

The Present Position of the Surgery of Malignant Growths. By CHARLES A. MORTON, F.R.C.S. .. .. .. .. .. 289

Household Syphilis (Œconomica), with Observations on Acquired Syphilis in Infants. By J. A. NIXON, B.A., M.B. Cantab., F.R.C.P. .. .. .. .. .. .. 301

Some Observations on Tea-Poisoning. By NEWMAN NEILD, M.B. Vict., M.R.C.P... .. .. .. .. .. .. 312

Cardiac Arrhythmia: Five Cases. By CAREY COOMBS, M.D. Lond., M.R.C.P. .. .. .. .. .. .. .. .. 321

Herpes and Brachial Neuritis. By G. MUNRO SMITH, M.R.C.S. .. 332

The Bristol Medical Library. By L. M. GRIFFITHS, M.R.C.S. Eng., L.R.C.P. Edin. .. .. .. .. .. .. .. 335

PROGRESS OF THE MEDICAL SCIENCES .. .. .. 67, 165, 345

REVIEWS OF BOOKS .. .. .. .. 80, 171, 256, 353

EDITORIAL NOTES .. .. .. .. .. 88, 180, 278, 366

NOTES ON PREPARATIONS FOR THE SICK .. 90, 185, 281, 373

THE LIBRARY OF THE BRISTOL MEDICO-CHIRURGICAL SOCIETY .. .. .. .. 93, 188, 283, 376

MEETINGS OF SOCIETIES .. .. .. .. .. 95, 191, 378

OBITUARY .. .. .. .. .. .. 98, 195, 284, 382

LOCAL MEDICAL NOTES .. .. .. .. 100, 199, 287, 383

### The Library of the Bristol Medico-Chirurgical Society.

*The following donations have been received since the publication of the List in December.*

*February 28th,* 1911.

J. A. Nixon, M.B. (1) .. .. .. .. 1 volume.
The Surgeon-General, United States Army (2) 1 ,,

Framed pictures have been presented by Mr. T. Carwardine, Mr. L. M. Griffiths, and Dr. James Swain.

### SEVENTY-NINTH LIST OF BOOKS.

The titles of books mentioned in previous lists are not repeated.

The figures in brackets refer to the figures after the names of the donors, and show by whom the volumes were presented. The books to which no such names are attached have either been bought from the Library Fund, or received through the *Journal.*

**Adam, C.** .. .. *Handbook of Treatment for Diseases of the Eye* (Trans. from second German edition by W. G. Sym and E. M. Lithgow) .. .. .. 1911

**Beddoe, J.** .. .. *Memories of Eighty Years* .. .. .. .. 1910

**Berkeley and V. Bonney, C.** *Gynæcological Surgery* .. .. .. 1911

**Bonney, C. Berkeley and V.** *Gynæcological Surgery* .. .. .. 1911

**Bresler, J.** .. .. *The Treatment of Syphilis by the Ehrlich-Hata Remedy* (Trans. by M. D. Eder) 2nd Ed. 1910

**Carpenter, G.** .. *Golden Rules for Diseases of Children* (Revised by E. B. Smith) .. .. .. .. 4th Ed. [1910]

*Catalogue* (*Index-*) *of the Library of the Surgeon-General's Office, United States Army,* 2nd Series. Vol. XV .. .. .. (2) 1910

**Clarke, W. B.** .. *Surgery of the Kidneys* .. .. .. .. .. .. 1911

**Crandon, L. R. G.** *Surgical After-treatment* .. .. .. .. .. .. 1910

**Despard, L. L.** .. *Textbook of Massage* .. .. .. .. .. .. 1911

**Donahoe, M. F.** .. *A Manual of Nursing* .. .. .. .. .. .. 1910

**Fishberg, M.** .. *The Jews* .. .. .. .. .. .. .. .. .. .. 1911

**Fordyce, A. D.** .. *The Hygiene of Infancy and Childhood* .. .. 1910

**Glaister, J.** .. .. *A Textbook of Public Health* .. .. 2nd Ed. 1910

**Green, C. E.** .. *The Cancer Problem* .. .. .. .. .. .. .. 1911

*Gynecology* (*Catechism Series*) .. .. .. .. .. .. .. .. .. [N.D.]

**Hartridge, G.** .. *Golden Rules of Ophthalmic Practice* 5th Ed. [1910]

**Hurry, J. B.** .. *Vicious Circles in Disease* .. .. .. .. .. 1911

*Infant and Child Mortality* .. .. .. .. .. .. .. .. .. .. ..

**Kenwood, H. R.** .. *Public Health Laboratory Work* .. 5th Ed. 1911

**Kenwood, L. C. Parkes and H. R.** *Hygiene and Public Health* 4th Ed. 1911

| | | | |
|---|---|---|---|
| **Lapage, C. P.** .. | *Feeblemindedness in Children of School Age* .. | | 1911 |
| **MacDonald, D. M.** | *Ambulance Examination Questions* | 4th Ed. | 1911 |
| **Marie, A.** [Ed.] .. | *Traité international de Psychologie pathologique* | Tome II | 1911 |
| **Martindale, W. H.** | *Organic Analysis Chart* .. .. .. .. .. .. | | 1910 |
| **Martindale and W. W. Westcott, W. H.** | *" Salvarsan "* (" 606 ") | | 1911 |
| **Martindale and W. W. Westcott, W. H.** | *The Extra Pharmacopœia* | 14th Ed. | 1910 |
| **Moore, B.** .. .. | *The Dawn of the Health Age* .. .. .. .. | | 1911 |
| **Moullin, C. M.** .. | *Enlargement of the Prostate* .. .. | 4th Ed. | 1911 |
| **Muir and J. Ritchie, R.** | *Manual of Bacteriology* .. .. | 5th Ed. | 1910 |
| **Parkes and H. R. Kenwood, L. C.** | *Hygiene and Public Health* | 4th Ed. | 1911 |
| **Rawling, L. B.** .. | *Landmarks and Surface Markings* | 4th Ed. | 1911 |
| **Rhodes, G.** [Ed.] | *Medicine and the Church* .. .. .. .. | | 1910 |
| **Ritchie, R. Muir and J.** | *Manual of Bacteriology* .. | 5th Ed. | 1910 |
| **Routh, A.** .. .. | *Cæsarean Section in Great Britain and Ireland* | | 1911 |
| **Sherlock, E. B.** .. | *The Feeble-Minded* .. .. .. .. .. .. .. | | 1911 |
| **Stevens, W. M.** .. | *Medical Diagnosis* .. .. .. .. .. .. .. | | 1910 |
| **Stitt, E. R.** .. .. | *Practical Bacteriology* .. .. | 2nd Ed. | 1911 |
| *Surgery (Catechism Series)* .. .. .. .. .. .. .. | | 2nd Ed. | [N.D.] |
| **Thomson, J. A.** .. | *Outlines of Zoology* .. .. .. .. | 5th Ed. | 1910 |
| **Vincent, R.** .. .. | *On Acute Intestinal Toxæmia in Infants* .. | | 1911 |
| **Westcott, W. H. Martindale and W. W.** | *" Salvarsan "* (" 606 ") | | 1911 |
| **Westcott, W. H. Martindale and W. W.** | *The Extra Pharmacopœia* | 14th Ed. | 1910 |
| **Whittaker, C. R.** | *A Manual of Surgical Anatomy* .. .. .. | | 1910 |
| **Williamson, R. T.** | *Diseases of the Spinal Cord* .. .. | 2nd Ed. | 1911 |
| **Wyllie, J.** .. .. | *Meningitis, Sinus Thrombosis and Abscess of the Brain* .. .. .. .. .. .. .. .. | | 1911 |

### TRANSACTIONS, REPORTS, JOURNALS, &c.

| | | |
|---|---|---|
| American Journal of the Medical Sciences, The .. | Vol. CXL | 1910 |
| American Journal of Obstetrics, The .. .. .. | Vol. LXI | 1910 |
| Archiv für klinische Chirurgie .. .. .. .. .. | Bd. XCI | 1910 |
| Berliner klinische Wochenschrift .. .. .. .. .. .. .. | | 1910 |
| British Medical Journal, The .. .. .. .. .. | Vol. II for | 1910 |
| Clinical Journal, The .. .. .. .. .. .. .. | Vol. XXXVI | 1910 |
| Deutsche Zeitschrift für Chirurgie .. .. .. | Bds. CII–CIV | 1910 |
| Edinburgh Medical Journal, The .. .. .. | N.S., Vol. V | 1910 |
| Glasgow Medical Journal, The .. .. .. .. | Vol. LXXIV | 1910 |
| Hospital, The .. .. .. .. .. .. .. .. .. | Vol. XLVIII | 1910 |
| Hospital Reports, Guy's .. .. .. .. .. .. | Vol. LXIV | 1910 |
| Infant and Child Mortality, L.G.B. Report on .. .. .. .. | | 1910 |
| Journal of the American Medical Association, The .. | Vol. LV | 1910 |
| Journal of Hygiene, The .. .. .. .. .. .. .. .. | Vol. IX | 1909 |
| Lancet, The .. .. .. .. .. .. .. .. .. .. .. | Vol. II for | 1910 |
| Library World, The .. .. .. .. .. .. .. .. | Vol. XII | 1910 |

| | | |
|---|---|---|
| Medical Chronicle, The | Vol. LII | 1910 |
| Medical Directory, The | | 1911 |
| Medical Record, The | Vols. LXXVII, LXXVIII | 1910 |
| Medical Review, The | Vol. XIII | 1910 |
| Minerva | (1) 1910–11 | 1911 |
| Münchener medizinische Wochenschrift | Bd. II for | 1910 |
| Practitioner, The | Vol. LXXXV | 1910 |
| Prescriber, The | Vol. IV | 1910 |
| Progressive Medicine | Vol. IV | 1910 |
| Quarterly Journal of Medicine, The | Vol. III | 1909–10 |
| Revue de Chirurgie | Bd. XLI | 1910 |
| Revue hebdomadaire de Laryngologie | Tome XXX, Vol. II | 1910 |
| Royal Society of Medicine, Proceedings of the | Vol. III | 1910 |
| West London Medical Journal | Vol. XV | 1910 |
| Wiener klinische Wochenschrift | | 1910 |
| Year-Book of Scientific and Learned Societies | | 1910 |

### MEETINGS OF SOCIETIES.

### Bristol Medico-Chirurgical Society.

### Local Medical Notes.

### UNIVERSITY OF BRISTOL.

**University Settlement.**—This scheme, to which we drew attention in our last number, has definitely come to a head, and the Committee have chosen a site for their first operations at Barton Hill. It will be a women's settlement, but men will help in the work, and will be under the organisation of the University, who will supply the workers for it.

### EXAMINATION RESULTS.

M.B., Ch.B. BRISTOL.—R. C. Clarke, F. P. Mackie, R. S. S. Statham, W. Reynolds. *Part I:* J. F. H. Morgan.

B.D.S. BRISTOL.—C. J. Kelsey.

M.D. LOND.—*Medicine:* Cecil Clarke.

CONJOINT BOARD OF LONDON.—*Anatomy and Physiology:* E. D. D. Davies. *Medicine:* W. W. Pratt, V. Pinnock. *Surgery:* S. Marle. *Midwifery:* G. F. Fawn.

M.R.C.S., L.R.C.P. LOND.—W. W. Pratt, S. Marle, M. W. Morrison.

L.D.S.—*Chemistry:* K. Batten, W. J. Milton, E. E. Wookey. *Physics:* E. E. Wookey. *Final* (*Part I*)*:* L. G. Kinnersley, C. V. Osborne, S. Saxton. *Part II:* F. C. Willows, Grantley Smith.

I.M.S.—E. S. Goss (*8th place*), J. F. H. Morgan (*9th place*), P. S. Connellan (*13th place*).

### APPOINTMENTS.

**University of Manchester.**—I. Walker Hall, M.D. Vict., has been appointed External Examiner in Pathology.

**Berkeley Hospital.**—Arthur John Awdry, L.R.C.P. Lond., M.R.C.S., and Walter Robert Awdry, M.B. Durh., M.R.C.S., have been appointed Honorary Medical Officers.

**Bristol Eye Hospital.**—The following appointments have been recently made :—*Consulting Surgeon :* Clements Hailes, M.D., F.R.C.S. Edin. *Surgeon :* Alexander Ogilvy, M.D. Durh., F.R.C.S.I. *Anæsthetist :* W. S. Vernon Stock, M.B., B.S. Lond., F.R.C.S. *Rhinologist :* A. J. M. Wright, M.B., B.S. Lond., F.R.C.S.

**Moretonhampstead Cottage Hospital.**—Alfred Coleridge, M.B., B.S. Lond., has been appointed Honorary Medical Officer.

**South Devon and East Cornwall Hospital, Plymouth.**—George F. Aldous, F.R.C.S. Edin., has been appointed Honorary Surgeon.

**Bradford Royal Infirmary.**—James Phillips, F.R.C.S. Edin., has been appointed Honorary Surgeon.

**Bristol Royal Infirmary.**—The following appointments have been made recently :—*Dental House Surgeon :* G. F. Fawn, L.D.S. *Medical Registrar :* C. W. J. Brasher, M.B., Ch.B. *Surgical Registrar :* A. J. Wright, M.B. Lond., F.R.C.S. *Honorary Dental Anæsthetist :* C. F. A. Hereford, M.R.C.S., L.R.C.P. Lond.

**Bristol General Hospital.**—B. J. Geakie, L.D.S., has been appointed Dental House Surgeon.

**Nineteenth International Congress of Medicine, London, 1913.**—Professor J. Michell Clarke has been appointed representative of the University of Bristol on the Executive Committee and a member of the Council of the Section of Neuro-pathology. P. Watson-Williams, M.D., has been appointed a member of the Council of the Section of Laryngology. J. M. Fortescue-Brickdale has been appointed a member of the Council of the Section of Pharmacology.

**Third International Congress of Laryngology, Berlin, 1911.**—Dr. P. Watson-Williams, President of the Section of Laryngology of the Royal Society of Medicine, has been appointed a representative of Great Britain and Ireland on the Executive Committee.

### BRISTOL.

**Athletic Field.**—Through the munificence of Mr. George A Wills the Athletic Union is now assured of an athletic ground within easy reach of the University. For this purpose he has given £4,000, which will give the Union the use of a twelve-acre field in Coombe Lane. The Council of the University has officially promised to support the scheme with an annual contribution towards its upkeep. The work of levelling and of erecting suitable dressing-rooms and pavilion will be put in hand immediately.

**"Bristol Nonesuch."**—The first number of the new University magazine has made its appearance, in a cover the colour known as "Bristol Red." It contains a portrait of the donor of the new athletic field, Mr. G. A. Wills, and information relating to the various student and other organisations of the University. All those interested in the University should obtain a copy of the first number. It will make its appearance three times annually, and the subscription is 1s. 9d.

**Bristol Dispensary.**—During 1910 the number of patients treated by this institution increased by 681 over the previous year. 688 midwifery notes were distributed, and medical assistance was sought for in 31 cases. The old building in North Street which was the dispensary of many years ago has been disposed of for £750.

**Bristol Eye Hospital.**—At the annual meeting, held recently, the report stated that 474 in-patients had been admitted, and 7,061 out-patients had attended at the hospital. The financial state of the hospital for the year was satisfactory, mainly owing to the proceeds from the recent fête, which freed the institution from debt, and enabled the Committee to establish a small nurses' home close to the hospital, the rooms which they had used in the building being given up to the patients' use.

**Clifton Dispensary.**—This institution during the past year treated 2,420 medical cases, 312 surgical cases, and 71 cases of midwifery. There was an adverse balance during the year of £8, in spite of an increase in subscriptions.

### BRIDGWATER.

At the annual meeting of the Bridgwater Hospital two memorial brasses erected in memory of two members of the honorary medical staff, the late Dr. W. L. Winterbotham and Dr. F. J. C. Parsons, were unveiled. A bust of the founder of the hospital, Dr. Jonathan Toogood, was also uncovered at the meeting.

### WESTON-SUPER-MARE.

Recently the new Isolation Block of the Weston-super-Mare Hospital was formally opened. It contains two wards for the accommodation of four patients and nurse's room.

### BURNHAM.

The scheme for providing a Cottage Hospital for Burnham and Highbridge is being well taken up, and the medical men of the neighbourhood are giving it their support, as such an institution is a desideratum.

### BATH.

The annual meeting of the Royal United Hospital was held recently under the presidency of the Mayor. The financial statement for 1910 showed that the deficit on the working expenses amounts to the sum of £1,838, in spite of rigid economy. The medical report was most satisfactory.

**The War against Consumption.**—From Brecon :

EDWARD VII MEMORIAL SCHEME FOR WALES.

Avaunt, insidious plague ! thy days are numbered
(For until late humanity has slumbered),
Who wroughtest havoc on the lungs of man,
Thy deadly germs we must resolve and can
Dispell, destroy, and stop their propagation
By many means, among them ventilation.
Of late we know that thou, consumptive bug,
Art (need I say ?) a denizen of fug ;
And if we can conform to open air,
That hot, close rooms become a thing most rare,
Why then thou deadly tubercle bacillus,
There is slight scope or power for you within us ;
And if by chance thou dost procure a hold,
Means for disarming thee are manifold.
The one and all important thing to grasp
Is thy detection e'er thou firmly clasp
The human lungs in thy retentive grip,
From which till recently one might not slip.
Now thy dire purpose to frustrate we should
Get out each day and not refrain from food,
Avoid as far as possible contagion,
Direct or indirect, from a relation ;
See that the teeth and gums in order stand,
'Gainst all disease the body be well manned.
Should chronic cough importunate appear,
Just seek advice and dissipate your fear.
The signs which point to quite early infection,
As loss of weight, must bring about detection ;
Expectoration, chronic cough and pain,
The breath being short, and you your food disdain,
With sweats at night of quite a large amount,
Why then methinks t'were well to take account ;
For these with other signs all tell the tale,
No use it is your prospect to bewail,
The boundless energy and spirits fail,
The hectic tinge shows well the doleful tale ;

The temper, manner, disposition, too,
All manifest the diagnosis true.
Now, once 'tis known that thou art of the smitten,
And Sans. are full or otherwise forbidden,
In thine own home, but under supervision,
It well becomes thy duty and thy mission,
To bear with calmness, and to persevere
The restful treatment we should prize so dear.
The objective, then, is food and rest and air,
Eat and digest, abstain from wear and tear ;
Free from excitement, exercise or strain,
The chest should never quickly move on pain
Of giving our bacillus further hold ;
And should the weather turn severe or cold,
When not unlikely you discomfort feel,
On no account the window casement seal,
But strike a match and let the fire glow,
And you yourself immune to cold will grow ;
Add further clothing should one feel inclined,
Acquire good books to occupy the mind.
'Gainst spitting, as is common, please refrain,
In France " Défense de crâcher " in the train
And on a poster planted everywhere
Was written large some thirteen years or near.
The sputum dries, the germs disseminate,
Infect their natural habitat and emanate
From other lungs, and let the white plague spread
An illness every mortal learns to dread.
Koch's method, now tho' modified, we use,
Tuberculin injections daren't refuse,
Combined with sanatorium life we find,
And most concerning this are of one mind,
That tho' each helps, combined they greatly tend
The blood resistances higher up to send ;
The tissues strengthened with new energy,
Enable man in time his lethargy
To overthrow and with new zeal arise,
Which but a little back would cause surprise.
And now to close I would just merely say,
To put a finish to this phthisic lay,
That we no more a willing host must be,
But show T.B. no lack of enmity ;
The fearful fight resume at any cost,
That man and his successors be not lost.

